# Environmentally-Friendly Materials in Wastewater Treatment

**DOI:** 10.3390/ma16186181

**Published:** 2023-09-13

**Authors:** Inga Zinicovscaia, Magdalena Balintova

**Affiliations:** 1 Department of Nuclear Physics, Joint Institute for Nuclear Research, Joliot-Curie Str., 6, 1419890 Dubna, Russia; 2 Department of Nuclear Physics, Horia Hulubei National Institute for R&D in Physics and Nuclear Engineering, 30 Reactorului Str. MG-6, 077125 Magurele, Romania; 3 Department of Environmental Engineering, Institute for Sustainable and Circular Construction, Faculty of Civil Engineering, Technical University of Kosice, Vysokoskolska 4, 042 00 Kosice, Slovakia; magdalena.balintova@tuke.sk

The intensive development of industry and agriculture caused by high population growth results in the release of large volumes of wastewater containing organic and inorganic pollutants into the environment. Careful treatment of wastewater is an essential step before its discharge into natural water bodies. Recently, a wide variety of physical, chemical, and biological approaches have been applied to wastewater treatment. Applied techniques have a number of advantages, but at the same time they are not without limitations. Among the disadvantages of applied techniques, it is worth noting their high cost and energy consumption, as well as the generation of hazardous waste, which can lead to secondary pollution. Thus, it is critically important to develop new, environmentally friendly technologies for wastewater treatment.

This Special Issue aimed to present the main research achievements in the development of materials and approaches for the removal of pollutants of different origins from wastewater. It provides a platform for scientists to present their original research on “Environmentally Friendly Materials in Wastewater Treatment”.

This Special Issue includes 11 original research papers related to the topic, which have been prepared by scientists from different countries. A brief overview of each paper, which we are honored to edit as Guest Editors, is presented below in order to highlight the recent progress in the application of environmentally friendly materials in wastewater purification.

Among the techniques applied for wastewater treatment, microbial technologies are considered an environmentally friendly alternative to existing methods, due to their cost-effectiveness, non-invasiveness, and environmental safety. Yushin and co-authors [1], for the first time, tested the applicability of the cyanobacteria *Arthospira platensis* for erbium ions removal from wastewater. Experiments with living (bioaccumulation) and dry (biosorption) biomasses were performed. The results show that erbium removal by dry *Arthospira platensis* depends on pH, time of contact, and temperature. The maximum biomass sorption capacity of 30 mg/g can be attained at pH 3.0 and room temperature after 3 min of sorbent interaction with the sorbate. *Arthospira platensis* living biomass showed a high accumulation capacity toward erbium ions (45–78%). The growth of the biomass in the presence of erbium ions did not affect biomass productivity or the content of proteins, carbohydrates, and pigments, however, it resulted in a decrease in the level of lipids. *Arthospira platensis* can be regarded as a safe and efficient bioremediator for erbium-contaminated wastewater treatment.

Often, the use of microorganisms, especially bacteria, for wastewater treatment is complicated by the formation of a biofilm that makes their accumulation capacity non-constant, and biofilm removal from the treated effluent is a difficult task. Zinicovscaia and co-authors [2] proposed the growth of the bacteria *Shewanella xiamenensis* biofilm on zeolite, which resulted in the formation of a mineral-organic sorbent. The obtained sorbent was applied for metal removal from complex zinc-containing effluents (synthetic and real). In batch systems, the maximum sorption capacity of the sorbent changed from 3.4 to 6.5 mg/g and was attained at a pH range of 3.0–6.0 within 60–150 min of sorbent–sorbate contact. In the case of a real industrial effluent, a maximum zinc removal of 85% was achieved at pH 6.0. To achieve high removal efficiency, the authors applied a two-step treatment scheme.

Besides biological adsorbents, mesoporous materials are of great interest for adsorption studies due to their high surface area, thermal and chemical stability, and eco-friendly characteristics. Two mesoporous materials, silica SBA-15 and titanosilicate ETS-10, were applied in [3] for indium removal from wastewater. It was found that the maximum sorption capacity of silica SBA-15 (2036 mg/g) was higher than that of titanosilicate ETS-10 (366 mg/g), mainly due to the hydroxylation of indium ions as well as the large diameter of silica SBA-15 pores. Both materials maintained high adsorption capacities during six adsorption and desorption cycles. The ORCA quantum chemistry program package applied to investigate interactions between the indium sulfate structure and surfaces of adsorbents allowed for the conclusion that three covalent bonds can be formed between indium atoms and oxygen atoms in the case of silica SBA-15, due to the presence of silanol groups on the surface. Cepan et al. [4] tested different adsorbents, magnetite, cobalt ferrite, zinc oxide, titanium dioxide, and mordenite zeolite, for phosphorous removal from wastewater. The highest removal efficiency of 96% was obtained for mordenite zeolite, followed by magnetite (88%), cobalt ferrite (80%), titanium dioxide (71%), and zinc oxide (51%). The maximum adsorption capacity of the tested materials computed from the Langmuir model changed in the following order: magnetite > zeolite > cobalt ferrite > titanium dioxide > zinc oxide. The authors recommended the reuse of the tested materials as fertilizers or for other organic or biological pollutant removal.

The increasing production and application of synthetic dyes resulted in widespread water contamination. Pipíška and co-authors [5] proposed the thermochemical conversion of waste wood chips and corn-cob biomass to biochars to obtain cheap and efficient adsorbents for erythrosine B and thioflavin T removal from wastewater. The maximum adsorption capacity of wood chips and corn cobs for erythrosine B was 12.7 mg/g and 1.5 mg/g, respectively. Both materials showed higher adsorption capacities for thioflavin T: 182 mg/g for wood chips and 44.6 mg/g for corn cobs. The authors showed that adsorption was only slightly affected by solution acidity and suggested that the main mechanisms of dye adsorption are π–π interactions, hydrogen bonding, pore filling, and interactions with mineral components. The obtained adsorbents are more suitable for the treatment of wastewater containing cationic dyes. The coloration of different products is often associated with the application of metal mordants, leading to the disposal of unfixed toxic metals into the environment after dyeing. The efficiency of zeolite 4A in two particle sizes and activated carbon for the removal of color, chemical oxygen demand, total organic carbon, and metals from wastewaters after natural wool dyeing was assessed in [6]. Applied adsorbents were able to reduce wastewater coloration considerably: activated carbon up to 78% and zeolite 4A up to 71%. Activated carbon proved to be very effective in the removal of chemical oxygen demand (up to 96%) and total organic carbon (up to 95%). At the same time, the best reduction in metallic ions (iron, copper, and aluminum), turbidity, and electrical conductivity was achieved using the 4A zeolite. The authors concluded that further studies are required in order to optimize the process of treating wastewater with a complex chemical composition.

The pollution of water resources by pharmaceuticals, including antibiotics, is alarming because of their adverse effects on microorganisms, aquatic animals, and human health through bioaccumulation in food chains. Lam and co-authors [7] used NiFe_2_O_4_@C composites, from a bimetallic-based metal-organic framework Ni-MIL-88B(Fe) fabricated using a solvothermal method, for the removal of ciprofloxacin (CFX) and tetracycline (TCC). The combination of magnetic components (e.g., NiFe_2_O_4_) with a porous carbon structure allowed for a higher antibiotic removal due to the high surface area of porous carbon and the excellent separation properties of the magnetic components. The composite produced at 900 °C possessed high adsorption capacities for both antibiotics, CFX and TCC. The optimal conditions for CFX adsorption were identified as a concentration of 40.0 mg/L, an adsorbent weight of 0.148 g/L, and pH 3.97. The TCC adsorption was the most efficient at a concentration of 23.93 mg/L, an adsorbent weight of 0.115 g/L, and pH 3.6. Under optimum conditions, the experimental adsorption capacities were 256.24 and 105.38 mg/g for CFX and TCC, respectively.

In the last two decades, there has been a growing interest in anaerobic technologies, which are perceived as competitive with conventional aerobic methods. Unfortunately, their main drawback is their low effectiveness in the removal of biogenic compounds, mainly phosphorus, which is the most important factor responsible for the eutrophication of natural aquatic ecosystems. The authors of [8] presented an active filling produced via microcellular extrusion technology, which contains admixtures of metals, minerals, or other elements. The authors determined its properties and showed its performance in anaerobic wastewater treatment. The modification of plasticized poly(vinyl chloride) (PVC) with the blowing agent and metal powders enabled them to obtain a porous extrudate with a modified physical structure. The influence of copper and iron admixtures on the properties of the obtained porous extrudate in terms of its functional properties was also examined. The addition of metal powders caused an increase in the extrudate density. The modification of PVC resulted in obtaining the highest porosity, amounting to 47.0%, and caused a 50% decrease in the tensile strength. The use of the filling in anaerobic rectors promoted chemical oxygen demand removal, intensified biogas production, and allowed for the elimination of 64.4–90.7% of phosphorus, depending on the type of wastewater and the applied technological parameters. Zieliński et al. [9] tested waste-derived, low-cost recycled filling (LCRF) for the treatment of real-world dairy wastewater in pilot-scale anaerobic reactors, as well as assessing how different digester loadings affect organic compound removal, nutrient take-up, biogas composition and yield, and the structure of the bacterial taxa. The LCRF was made from commercially available waste (leftover mixed plastics from metal recycling of scrap wiring and electrical systems, mostly junked cars). The waste plastics consisted of various insulating materials (normal rubber, silicone rubber, halogen free material, cross-linked polyethylene, organohalogen material, softened PVC coating, and heat-resistant softened PVC coating), as well as iron, copper, and aluminum. Anaerobic digestion (AD) performance was compared against lightweight expanded clay aggregate filling (LECAF) reactors. The addition of LCRF was found to increase both chemical oxygen (86.1–92.8%) and total phosphorous (22.1% to 36.9%) removal from the wastewater. Across all tested pollutant loads, the LCRF ensured near-neutral pH and stabilized the structure of the anaerobic microbe community (including Archaea). This allowed efficient biogas production and high methane content in the LCRF reactors, peaking at 0.35 m^3^/kg COD_removed_ and 68.2% (respectively) in the best-performing variant. The LECAF anaerobic reactors had little success in removing total phosphorous, with removal rates falling within a narrow range, from 3.6% to 5.7%. Across all of the experimental variants, the LCRF reactors proved superior in terms of digestion performance, with biogas production ranging from 0.67 m^3^/d to 1.94 m^3^/d (depending on the organic load rate (ORL)). LCRF reactors also produced more methane in the biogas at the two highest OLRs, with fractions of 68.2% and 67.3%.

Pollution of surface water results in the contamination of sediments and can potentially have an adverse impact on receptors including benthic and water-column invertebrates, fish, wildlife, plants, and human populations. Urbancl et al. [10] studied the process of selected heavy metal (Cu(II), Ni(II), and Cr(VI)) removal from polluted river sediments using a zero-waste technological solution. The proposed technology consists of sediment washing and purification of the wastewater produced as a by-product. EDTA and citric acid were used for the extraction of elements from the studied samples. Washing with citric acid showed better performance compared to EDTA; 60% of Cu(II) was removed with EDTA compared to 80% removed with citric acid. Next, a solution for the hybrid removal of heavy metals from river sediments was developed based on the laboratory experiment results of washing with citric acid. The working process of sediment treatment was planned in two lines. The treatment of 43,000 tons of waste sediment was carried out for 345 days. After separation, the wastewater produced as a by-product was treated using natural clay. Clay, composed mainly of silicon and aluminum oxides, proved to be a satisfactory choice, allowing for the removal of 99% of Cu(II) and 80% of Cr(VI), while the concentration of Ni(II) remained below the limit of 1 mg/kg d.m. due to the adsorption process. The presented results show the potential of the zero-waste hybrid system for sediment remediation.

The discharge and leakage of oily waste materials into water resources, including seawater, groundwater, and freshwater, has become a significant problem in recent decades. Therefore, there is a crucial need to develop materials and technologies for separating oil and water (O–W). In work [11], the fabrication of a robust carbon quantum dots (CQDs)-based superhydrophobic (S.P) membrane for oil/water separation was reported. The CQDs, prepared using banana leaves via the hydrothermal method, exhibited a circular shape with an average size of 4.4 nm and a crystalline structure. Furthermore, the BET analysis determined that the prepared CQDs had a specific surface area of 845 m^2^/g, a pore volume of 0.33 cm^3^/g, and a mean pore diameter of 1.62 nm. The developed superhydrophobic textile fabric membrane exhibited excellent S.P properties, with a high water contact angle of 163 degrees and a low water sliding angle of 1 degree. The membrane also showed a good oil absorption capacity, separation efficiency, and flux rate towards three different oils, namely n-hexane, petroleum ether, and silicone oil. The results highlighted the effectiveness of the superhydrophobic membrane for oil/water separation, which is crucial in the development of efficient and tailored separation materials for specific oil/water separation applications. The membrane also demonstrated good mechanical and chemical stability, with the ability to withstand abrasion and immersion in solutions of different pH values for varying immersion times.
